# Supplementary material for: Identification of photoperiod-induced specific miRNAs in the adrenal glands of Sunite sheep (Ovis aries)
Source: Front Vet Sci. 2022 Jul 22;9:888207. doi: 10.3389/fvets.2022.888207 (PMC9354845; doi:10.3389/fvets.2022.888207)
Supplement: Supplementary file 1 [file Data_Sheet_1.ZIP › Supplementary file/miRDeep2 file.zip!/miRDeep2 file/Novel_Structure/SP42A1/Novel_583.pdf]

Provisional ID	:	NC_019459.2_3167
Score total	:	2.3
Score for star read(s)	:	-1.3
Score for read counts	:	0
Score for mfe	:	2
Score for randfold	:	1.6
Score for cons. seed	:	
Total read count	:	18
Mature read count	:	18
Loop read count	:	0
Star read count	:	0

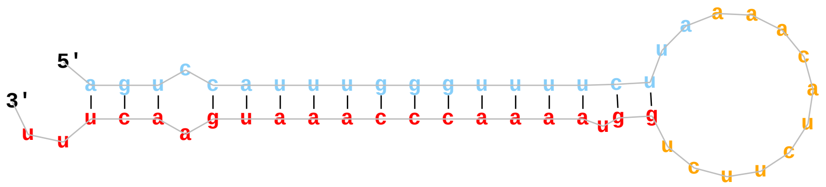

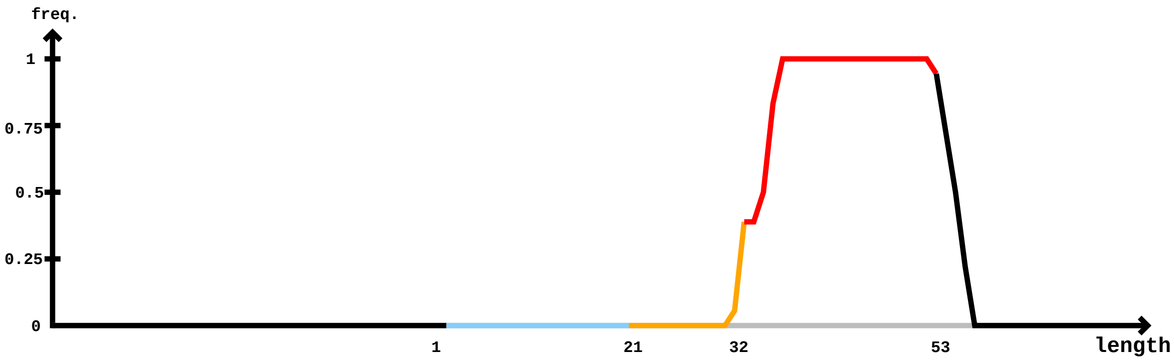


```
                                                Star                     Mature
5'- gcuuuaauauuauugggugaaauauuggguuggccaaaaaguccauuuggguuuucuuaaaacaucuucugguaaaacccaaaugaacuuuuuuuggccaauccaauauuuu   -3'    exp
    ..................((((((((((((((((((((((((.((((((((((((((.............)).))))))))))))).)))..)))))))))))))))))))))    reads      mm         sample
    .....................................................................uggAaaaacccaaaugaacuu......................    1          1          seq
    ......................................................................ggAaaaacccaaaugaacuuu.....................    5          1          seq
    ......................................................................gguaaaacccaaaugaacuuuu....................    1          0          seq
    ........................................................................Aaaaacccaaaugaacuuuu....................    2          1          seq
    .........................................................................aaaacccaaaugaacuuuuu...................    4          0          seq
    .........................................................................aaaacccaaaugaacuuuuuA..................    2          1          seq
    ..........................................................................aaacccaaaugaacuuuuu...................    1          0          seq
    ..........................................................................aaacccaaaugaacuuuuuA..................    1          1          seq
    ..........................................................................aaaUccaaaugaacuuuuuu..................    1          1          seq
```
